# Supplementary material for: Changes in sea floor productivity are crucial to understanding the impact of climate change in temperate coastal ecosystems according to a new size-based model
Source: PLoS Biol. 2023 Dec 11;21(12):e3002392. doi: 10.1371/journal.pbio.3002392 (PMC10712853; doi:10.1371/journal.pbio.3002392)
Supplement: Attachment — Submitted filename: TasmModel_ResponseToReview.docx [file pbio.3002392.s002.docx]

Dear Editors

We are submitting a revised version of the manuscript “Changes in benthic and pelagic production interact with warming to drive responses to climate change in a temperate coastal ecosystem” now renamed to “A new size-based model highlights the importance of benthic production changes for understanding climate change impacts on a temperate coastal ecosystem” number PBIOLOGY-D-22-01984R1 for your consideration to be published in PLOS Biology. We thank you for the opportunity to revise the manuscript and to clarify what we believe were misunderstandings about our goals and study design. The two reviews provided useful feedback and we believe that the revised manuscript is much improved as a result. We have now addressed the suggestion from reviewer 1 and have completely rewritten the discussion to more clearly highlight the novelty and implications of this study, as well as to expand on the limitations and future work needed.

As we understand, there are two main aspects that needed further clarification: 1) the representation of warming effects in the model, 2) the novelty and general importance of this study. We first provide a general response to these points and then address reviewer comments in detail.

*1) The representation of warming effects in the model systems*

We agree with both reviewers that warming is affecting ecosystems in multiple and complex ways and we only explore a small fraction of them. Specifically, we looked at five main effects of warming on marine life: i) acceleration of physiological rates, ii-iii) changes in the overall abundance of plankton or benthos resources (separately), iv-v) changes in the size composition of plankton or benthos resource (separately), and interactions of these five effects with species and trophic groups. Simulation results were analysed in a three-way ANOVA to account for possible three way interactions of physiology : resource change : different responses in different trophic groups (we note that such ANOVA analyses of a multi-species model scenarios were possible only due to our novel approach to parameter uncertainty, which we elaborate further in point 2 below). While this representation of warming effects is clearly simplified, in our view, a three way ANOVA is the maximum level of complexity that can be tackled in a single study of this kind, regardless of whether based on a modelling or experimental approach. We agree and acknowledge the many other ways in which warming affects marine ecosystems. Yet, as we expect the reviewers to appreciate, the complexities of real-world changes with warming are impossible to replicate or predict, and we see this study as providing insights into the potential effects of a few important changes.

The comment about not including many important aspects (responded to above) is at odds with the suggestion by reviewer 2 that our model has too many parameters: “*Give the shear number of parameter combinations (~30), it's unclear to me that this is valuable exercise because with so many components…*”. Adding further processes to the model will greatly increase the number of parameters. Moreover, while we agree that real physiological temperature responses are more complex, we do not have scientific consensus about the direction and magnitude of changes in different traits. Some studies suggest that intake rates will increase faster, others suggest that metabolism will increase faster. For example, (Audzijonyte *et al.*, 2022) used an individual fish growth model to explore outcomes of 42 realistic physiological response scenarios. All these 42 scenarios are backed up by literature. To put this into perspective, we are exploring 18 scenarios in our current study (9 resource change scenarios combined with 2 physiological change scenarios), which reviewer 2 suggests is too many (“*with enough parameters and scenarios, models can make so many predictions*”). If we were to consider these 9 resource change scenarios interacting with 42 possible options for physiological responses evaluated in Audzijonyte et al 2022, and for each of the 17 model species we would have 6426 scenarios.

The assumption that physiological responses are consistent across species allows us to explore how species interactions lead to different warming effects on biomass and size. For example, physiological changes had largest effects on predators and least impact on herbivores. If our model assumed different sensitivities across species, such a decomposition of responses would be impossible, and the model would not improve our understanding about the underlying processes. This is where we respectfully disagree with the reviewer’s 2 statement: “*To me, this is a good example of where we don't have the data to make realistic useful models to attack this problem but, with enough parameters and scenarios, models can make so many predictions, that they look like they are capturing the process, when they are not*”. We firmly believe the complex problem of how climate change affects marine ecosystems should still be tackled and that multi-species models, like the one used here, provide a useful tool to improve our understanding behind the processes and their interactions. Rather than making claims about what *will* happen in this system under a given climate response, we are using the study to learn how outcomes may vary with individual components. Importantly, we are careful about our wording, to make assumptions and the intentions of the study clear (also, see our further thoughts about physiological responses at the end of this letter).

A multi-species mechanistic model such as ours does include numerous parameters, and some researchers may have a fundamental opposition to such models. However, while we respect this opinion (shared to varying degrees by some of the co-authors on this paper), we believe that the aim of the study and application of the model is of critical importance. For example, fisheries stock assessment models (which are usually single species, but include many uncertain parameters) are applied to make management decisions, with their results intended to reflect the status of the underlying system. In our case, the model is designed to understand different dependencies in the system and potential knock-on effects of interactions. In other words, we seek to learn about processes in the system, rather than quantify the final net outcome. Many multi-species models are published in high impact scientific journals, including those that study community response to climate change. See, for example, the diversity of models summarised in the Fisheries and Marine Ecosystem Model Intercomparison Project (FishMIP) and published in highly cited journals (e.g. Lotze et al., 2019; Tittensor et al., 2021; Heneghan et al., 2021). Compared to many other widely used size or age structured marine multispecies models (e.g. Atlantis, OSMOSE), size based models in fact have relatively few parameters (e.g. Andersen 2019). Size based approaches offer a great opportunity to synthesise many complex processes into key community characteristics (Heneghan *et al.*, 2019). Given the increasing use of multi-species models for climate change predictions and policy making (especially in the light of the UN Decade of Ocean Science) it is imperative that we aim to improve their scientific rigour, which in our case involves rigorous and quantitative comparison of alternative warming impacts (see also e.g. Steenbeek et al., 2021). We aim for maximum transparency and replicability, with detailed supplementary material and all our code for model development, calibration and scenario runs available on a public GitHub repository.

Finally, in response to the reviewer comment “*this is a good example of where we don’t have the data to make realistic useful models*”, we would like to emphasise that our study does not attempt to make accurate predictions of observed changes, but only a theoretical exploration of how different climate driven processes interact. We do not aim to have an accurate model, but a useful model representing key processes of the ecosystems. To make this point clearer, we have now removed data points about empirical biomass and mean size changes over the last 30 years in Figs 2 and 3, because they seem to give a wrong impression that we were aiming to match them. We state that our scenarios do *not* represent real changes that occurred in the ecosystem, simply because we only simulated changes in plankton *or* benthos resource separately, when in reality both resources must have changed simultaneously. We have attempted to emphasise this further. A separate study would be needed to explore this interaction of relative plankton and benthos changes. This is why we developed and provide a shinyR app tool, where readers can explore these different combinations for themselves (which we believe is a novel and highly transparent approach to multi-species model outputs). Yet, it is also important to state that the model was calibrated with extensive field data.

In summary, we believe that our approach to model physiological responses is necessarily simplified and general, but justified for the broad question addressed here – relative importance of physiology versus food availability in a temperate coastal fish community with multiple primary production pathways, explored using a novel parameter uncertainty evaluation approach. We hope that this study will be used as a basis for further research, of which the most urgent one would be to assess the relative importance of physiological versus life-history responses. The revised paper now includes a longer discussion presenting the most pressing further questions that should be studied to improve our understanding of marine ecosystem responses to warming.

*2) The novelty and a general importance of this study*

Reviewer 2 suggests that “*The modelling is fairly straightforward, based on previous approaches used by this group of researchers and others*”. This is contrasted by Reviewer 1: “*I liked the factorial design for assessing parameter uncertainty; using it to find reasonable potential results without the need of catch data was very clever. This is very significant because it enables people to construct credible multispecies size spectrum models for data-poor communities or for future climates, which was very challenging, if not impossible, before. The authors’ effort to pursue ecological realism is detailed and impressive. Overall, I found this paper to be very illuminating*”. We would like to address Reviewer 2 criticism about the fact that the modelling is straightforward and based on previous approaches. It is true that we use a size-based modelling framework that has previously been applied in other studies. However, the framework itself is a methodological tool, which does not negate the novelty of this study. Many novel studies use well established tools (matrix models, general linear mixed effect models, age-structured models). Despite using a popular tool, we believe that this study delivers four important advances to the field:

1) The high potential importance of benthic primary productivity changes in determining shallow water aquatic ecosystem responses to climate change. Global ocean climate climate-biogeochemical models and most studies to date focus only on primary plankton production and zooplankton (Bryndum‐Buchholz *et al.*, 2019; Brandao *et al.*, 2021; Tittensor *et al.*, 2021), since they focus on shelf and offshore ecosystems and use data from global satellite observations and chlorophyll *a* models. In this study we show that for coastal marine ecosystems, which directly or indirectly sustain nearly one billion people, it is the benthic production pathway that has the largest impact on fish biomasses and yields. A study by Edgar et al. (2023), just published in Nature, assessed long term trends in coastal species’ populations around Australia and found that temperate reef invertebrates are the most rapidly declining group, which could potentially also affect fisheries yields. Yet, the Edgar et al. (2023) study also highlights that currently we have **almost no data on how benthic production is changing in response to warming**. Our modelling analysis, demonstrating the importance of benthic production for coastal ecosystems, highlights an urgent need to address this knowledge gap in order to improve our predictions about climate impacts on coastal and shallow freshwater ecosystems.
2) A new, simple but powerful approach to model multiple size structured background resources, which enables application of size-based models to coastal and freshwater ecosystems with distinct energy or resource production pathways (pelagic, benthic). This is significant and will be widely used in future research, coastal and freshwater community and ecosystem models, as demonstrated in highly attended international online courses (<https://course.mizer.sizespectrum.org/>).
3) Novel assessment of parameter uncertainty using a method similar to Approximate Bayesian Computation, and the calibration of a multispecies model to a range of ecological criteria. As reviewer 1 points out “*This is very significant because it enables people to construct credible multispecies size spectrum models for data-poor communities or for future climates, which was very challenging, if not impossible, before*”. In strict Bayesian uncertainty evaluation, tens or hundreds of millions of parameter combinations are sampled and evaluated using a likelihood function. Because we cannot construct a likelihood function for a complex model, we sample 2.2 million parameter combinations and evaluate them against ecological criteria, to retain only those that satisfy ecological realism in the baseline scenario. We then use these parameter combinations to assess the uncertainty of ecosystem responses to modelled climate change scenarios. As reviewer 1 identifies, this was not done before and is a novel and powerful way to add uncertainty evaluation into ecological models. To date many multi-species models are calibrated only to fisheries catch time series (as a proxy for species abundance) and sometime catch size composition data (Spence *et al.*, 2016; Oliveros-Ramos *et al.*, 2017; Lindmark *et al.*, 2021), even though there is knowledge about a much larger range of ecological processes. The need for such ecological realism, where “models produce right outputs for right reasons” is well recognised (Steenbeek *et al.*, 2021), but is rarely incorporated into model calibration. Here we implement a novel and relatively simple approach to assess model outputs against multiple ecological criteria and incorporate the resultant parameter uncertainty in model scenario comparisons. In our view, it is important that assessment of model scenarios includes uncertainty ranges, even though it is rarely done in multispecies models due to a lack of widely accepted protocol. We hope our approach will be useful for further urgently needed improvements in uncertainty evaluation of complex models, by helping to identify patterns for parameter selection in machine learning based approaches, defining informative priors for Bayesian approaches or improving the ways to sample a complex parameter space.
4) This study also introduces other methodological advances in ecosystem modelling, such as a supplementary user-friendly R Shiny application to explore model outputs in a transparent way, detailed and novel parameter selection approach for data poor fish species, and a statistical ANOVA-style design to compare alternative simulation scenarios. We believe this study is one of the most transparent ones available to date, aiming to introduce assumptions, strengths and weaknesses of size-based modelling to a broad audience. This is recognised by Reviewer 1: “*Lastly, I also wanted to highlight that this paper had one of the most thorough and useful Supplementary Materials I’ve ever read*”.

In summary, while we are using a size-based model calibrated for a real coastal ecosystem, our goal is more theoretical, aiming to understand relative importance of benthic, pelagic and physiological changes to coastal communities powered by distinct primary production pathways. We have now modified the discussion to further highlight the advances listed above.

Below we provide detailed response to reviewer comments.

*Reviewer #1:*

*This paper used a modelling approach to couple changes in planktonic and benthic production and warming effects on physiological processes of individuals and explored how these two things might, independently and jointly, alter food web dynamics in an Australian costal community. In doing so, the authors implemented well established size-spectrum models by explicitly separating the planktonic and benthic resource pathways and introduced a novel way of calibrating the model without using observed biomass or catch data. The implementation of the two resource pathways is also available as an R package. The main finding of this paper was that changes in resource dynamics generally had much stronger effects on species yields and biomasses than did those due to warming-related effects on physiology. Planktivores and benthivores were impacted more by changes in planktonic and benthic resources, respectively, and these impacts cascaded through the food web.
I liked the factorial design for assessing parameter uncertainty; using it to find reasonable potential results without the need of catch data was very clever. This is very significant because it enables people to construct credible multispecies size spectrum models for data-poor communities or for future climates, which was very challenging, if not impossible, before. The authors’ effort to pursue ecological realism is detailed and impressive. Overall, I found this paper to be very illuminating.*

We thank the reviewer for the positive feedback.

*As one of the evaluation criteria, the only place that this paper could benefit from additional data is a broader consideration of warming effects. This paper took into account how warming would impact species’ metabolic rate, search rate during foraging, intake rate, and predation-unrelated mortality. Even though none of these impacts is trivial, there are other warming consequences that could be at least equally important, such as changes in key life-history traits and shifts in species distribution, both of which can influence the modelling outcomes. I am not suggesting a total overhaul of the analyses, but it would be informative if there could be more discussions on whether (and how) this might have been behind some of the notable discrepancies between predicted and observed species biomasses (the authors did touch on these aspects in the Discussion, but only briefly).*

Yes, we fully agree that more detailed discussion would be useful, and we have now rewritten and expanded the discussion. We emphasise here again, however, that we were not attempting to match observed biomass or mean size changes with this model, as even our resource change scenarios were simplified (the observed biomass points are now removed from the main figure).

*Lastly, I also wanted to highlight that this paper had one of the most thorough and useful Supplementary Materials I’ve ever read. It would be even nicer if the authors could refer to specific sections of the SM in the main text.*

We appreciate this comment, especially considering that the lead author has put over 700 hours into the study, with the goal of providing a clear and transparent description of key assumptions and processes used in size-based models. We also followed the suggestion and improved the referencing to the supplementary material (and fixed incorrect referencing to supplementary figures listed below, apologies for that).

*I also listed several more minor comments below, most of which relate to methodological clarity. I hope my comments were helpful. I thank the authors for producing such an enjoyable and informative paper.*
*Minor comments*
*Line 159-162, Table 1 and Table S3. I like the approach of including a generalized large predator to account for their often-underestimated abundance, but how did the authors decide on the values for maximum weight, weight at maturity, and fishing mortality for this group? A maximum weight of 5 kg seems rather small for representing large predators. The choice of these values likely won’t impact the results dramatically, but some explanations/justifications may be necessary here.*

It is a somewhat arbitrary decision, as the “predator” is representing the pool of predators ranging from fish such as mackerel, various tunas, birds, seals. Many models do not represent such ‘external’ predation at all, but we felt it important to include, especially for our temperate coastal ecosystem, where the larger predators tend to operate in a system that expands beyond shallow coastal reefs (i.e. they have larger ranges and are more mobile). Potentially the predator could be larger, but it could have a large impact on the model species dynamics, so we assumed an intermediate size predator. We have now provided a short detail about the maximum size selection of this species: “This general predator was assumed to grow to a maximum of 5 kg, which is an approximate average across birds, commonly observed mackerel and other medium sized pelagic fish species (maximum size of 2-3kg), but also occasional sharks and mammals”.

*Figure S3 was supposed to show species’ responses to fishing based on their size, but that was not what was shown. It also looked like the references to the supplementary figures in the main text were off by one after Fig S2.
Table S5. The authors mentioned in the Supplement that the availability of the three resources to each species were given in Table S5, but I didn’t see this information there. Maybe the authors accidentally left it out?*

Thank you, this is now fixed. Figure S3 was eventually deleted as it was from the book by Andersen 2019 (now referenced in the Supplement section 1.5). Availability of the three resources is given in Table S4, columns aP, aB, aA (now correctly referenced). We believe that references to all supplementary figures and tables should now be correct.

*Line 402-406. It was not clear to me how the number of 1.8e6 was obtained from exploring two possible values of 37 variables (0.5 x and 2 x the original values). I intuitively thought that there would be a total of 2^37 parameter combinations, but this would produce a much larger number (~1.4e11). How did the authors decide which 1.8e6 combinations to sample? The same goes for the additional 0.4e6 samples (0.8 x and 1.2 x the original values). It might be worth adding one short sentence to explain this better.*

We have now explained this further and hopefully make it clearer (line 423): “To do this, for the 37 explored parameters, we generated 1.8 x10^6^ possible parameter combinations by sampling these 37 parameters from two-times larger space for each parameter; specifically each time the vector of the 37 parameters was multiplied by a random uniform vector ranging from 0.5 to 2, which means that each parameter could take any random value that was up to two times smaller or larger of its original value. A vast majority of these parameter combinations produced unrealistic model outcomes in terms of species relative abundances (some species went extinct). Therefore, to explore the smaller space around the original parameter values, we additionally sampled 0.4 x10^6^ parameter combinations, but this time multiplied the parameter vector by a random uniform vector ranging from 0.8 to 1.2 (i.e., parameter values were allowed to vary by 20%).”

*Line 417. I like the author’s approach of filtering out inappropriate parameter combinations by imposing extra fishing mortality. However, the authors seemed to have made several subjective choices regarding the threshold of responses beyond which parameters would be considered unrealistic (criteria 1-4: 0.33-3 x for fishing lobsters, 0.67 – 1.5x for fishing sea urchins, etc). I am not questioning these choices per se, but it would be helpful if the authors could explain their rationale behind these choices. It would help readers who would like to use a similar approach to decide whether they should use the same thresholds for their systems or, if not, how they should modify them.*

We used general size theory predictions about how much change in biomass is expected for species with different sizes and fishing resilience (Andersen 2019), as well as expert knowledge about the biomass levels of these species. The criteria are somewhat arbitrary, and we applied reasonably broad values. This is now explained in further details in the main text (line 436) and the supplement section 1.6 (second paragraph on the page 19).

*Reviewer #2:*

*This is a dense, parameter-rich exploration of how pelagic and benthic communities might change. The modelling is fairly straightforward, based on previous approaches used by this group of researchers and others. The model makes many predictions, depending on the scenario and inevitably, with so many predictions, some match real world changes. Give the shear number of parameter combinations (~30), it’s unclear to me that this is valuable exercise because with so many components, it’s near impossible for a model not to make some predictions that reflect reality.*

As we have tried to explain above, our study does not aim to match real observed changes in biomass or mean sizes. As we mention in the general response above, the goal of this study is broader than analysing one specific ecosystem, because we aim to understand relative importance of benthic, pelagic and physiological changes to coastal communities powered by distinct primary production pathways. To avoid this confusion, we now move figures with empirical observations to supplements and do not provide them in the main study.

*Yet with all of these parameters, the authors then take the oversimplified approach of assuming that all temperature-dependent processes have the identical temperature dependencies – an old-fashioned approach that has repeatedly been debunked since it was first proposed. To me, that approach is inadequate in this specific work for several reasons. First, we know that different processes have very different temperature dependencies. Second, we know that slight mismatches in temperature dependencies yield very different net outcomes from opposing processes. Finally, as the authors mention, these temperature dependencies themselves change with temperature through both evolution and plasticity. All in all, then for a paper that seeks to explore how warming affects function, the actual warming component is massively overly simplified – reduced to uniform rate functions.*

Thank you for the criticism and we agree that our representation of physiological responses is simplified. We have tried to address these points in the general response above. However, including different temperature dependencies and their acclimation and adaptation would require a vast number of additional parameters and intractable model complexity. Moreover, it would not allow us to assess how species interactions might be modifying outcomes of individual species physiological responses to temperature, and how physiological responses interact with changes in food abundance. The core question in our study was to determine relative effects of benthic vs pelagic production changes in a complex food web, not the effects of different types of temperature forcing. This is the reason we have used a simplified “default” approach to capturing temperature in line with models being used in large international projects, including FishMIP, rather than not including temperature effects at all. Even this simplified approach can reveal intricate responses. Finally, we emphasise that warming effects include changes in resources, and we have modified the text throughout the manuscript to make this clearer. See also the footnote at the end of this document for further thoughts about acclimation of physiological rates to temperature changes.

*Meanwhile, the authors explore such a massive range of scenarios that some congruence with reality is inevitable but not informative.*

We are exploring 18 scenarios, which is quite a lot, but something that can be handled and analysed in a three-way ANOVA framework. Each of these 18 scenarios are run with 29 parameter combinations, leading to 522 final simulations. It is important to note here that we are NOT looking for congruence with reality in some of these 522 simulations (and have modified Figures 2 and 3 to remove field data to avoid confusion). The 29 parameter combinations for each climate change scenario give a wide variation in responses and we are looking for how 18 scenarios differ given ALL of the likely parameter combinations. This is not unlike any other uncertainty evaluation. However, perhaps by the “massive range” the reviewer means the 2.2 million parameter combinations that were used in the parameter uncertainty evaluation process? These are not really scenarios, but sampling of parameter space, as done in a Bayesian analysis by sampling millions of parameter combinations through MCMC algorithm. We are using something similar to Approximate Bayesian Computation, where instead of evaluating model outputs against a specific likelihood function, we remove parameter combinations that do not fit a set of ecological criteria. In this way we are capturing some of the uncertainties surrounding parameters values - which are constrained by the data using the ABC method. We believe this approach provides an important improvement to current, often very limited, multi-species model parameter uncertainty evaluations, although naturally there is plenty of room for improvement, which we hope our study will encourage.

*In other words, because data are lacking on the key drivers of warming effects, the authors make strong, outdated assumptions about uniform temperature dependence while exploring so many different input scenarios that the model predictions include (among many others) responses that are similar to observations.*

We believe we have addressed this statement above, but we emphasise again that in our exploration of 18 scenarios we are not attempting to match any predictions to real observations, because these scenarios are clearly simplified (like the model itself). The 18 scenarios are used for theoretical exploration about relative importance of physiological, plankton and benthos changes in coastal ecosystems.

*To me, this is a good example of where we don’t have the data to make realistic useful models to attack this problem but, with enough parameters and scenarios, models can make so many predictions, that they look like they are capturing the process, when they are not. I’m very sorry I cannot be more positive but I don’t think this model achieves the ambitious goals that it sets itself because we simply lack the data we need to meaningfully model these processes.*

We respect the reviewer’s position on multi-species models and using them to better understand climate change impacts in marine ecosystems. Complex multi-species models often attract criticism that they are too complex (we explore uncertainty in 37 parameters) and at the same time not complex enough (temperature processes are too simplified, evolution and species redistribution not included). Nevertheless, there is a large scientific community that believes that multi-species models are essential to improve our understanding about ecosystems, because (a) we cannot make extrapolations from simple individual or population models defined by a few parameters; and (b) because observations of the real world can be affected by so many unknown correlations and causative processes that it can be impossible to tease apart which underlying processes are acting in which direction. Size based models offer an important middle ground, because they are based on strong theory to derive species parameters and their interactions, which constraints possible parameter values. We agree that our model is unlikely to make accurate predictions, but we believe it will be useful to explore how changes in the abundance and size structure of food availability interact with temperature driven physiological changes to affect fish communities living in coastal and freshwater ecosystems.

We hope the reviewer can appreciate the benefits of the approach, and see from R1s response, that not all scientists in the field share this view. We have attempted to be clearer about our goals in using the approach we have, but welcome a chance to further clarify the text, if the reviewer has suggestions which will help address their concerns.

**References**

Audzijonyte, A., Jakubavičiūtė, E., Lindmark, M. & Richards, S.A. (2022) Mechanistic temperature-size rule explanation should reconcile physiological and mortality responses to temperature. The Biological Bulletin, 243, 220-238.

Brandao, M.C., Benedetti, F., Martini, S., Soviadan, Y.D., Irisson, J.O., Romagnan, J.B., Elineau, A., Desnos, C., Jalabert, L., Freire, A.S., Picheral, M., Guidi, L., Gorsky, G., Bowler, C., Karp-Boss, L., Henry, N., de Vargas, C., Sullivan, M.B., Tara Oceans Consortium, C., Stemmann, L. & Lombard, F. (2021) Macroscale patterns of oceanic zooplankton composition and size structure. Sci Rep, 11, 15714.

Bryndum‐Buchholz, A., Tittensor, D.P., Blanchard, J.L., Cheung, W.W., Coll, M., Galbraith, E.D., Jennings, S., Maury, O. & Lotze, H.K. (2019) Twenty‐first‐century climate change impacts on marine animal biomass and ecosystem structure across ocean basins. Global change biology, 25, 459-472.

Edgar, G.J., Stuart-Smith, R.D., Heather, F.J., Barrett, N.S., Turak, E., Sweatman, H., Emslie, M.J., Brock, D.J., Hicks, J., French, B., Baker, S.C., Howe, S.A., Jordan, A., Knott, N.A., Mooney, P., Cooper, A.T., Oh, E.S., Soler, G.A., Mellin, C., Ling, S.D., Dunic, J.C., Turnbull, J.W., Day, P.B., Larkin, M.F., Seroussi, Y., Stuart-Smith, J., Clausius, E., Davis, T.R., Shields, J., Shields, D., Johnson, O.J., Fuchs, Y.H., Denis-Roy, L., Jones, T., Bates, A.E., 2023. Continent-wide declines in shallow reef life over a decade of ocean warming. Nature, 615, 858-865.

Heneghan, R.F., Hatton, I.A. & Galbraith, E.D. (2019) Climate change impacts on marine ecosystems through the lens of the size spectrum. Emerging Topics in Life Sciences, 3, 233-243.

Heneghan, R.F., Galbraith, E., Blanchard, J.L., Harrison, C., Barrier, N., Bulman, C., Cheung, W., Coll, M., Eddy, T.D. & Erauskin-Extramiana, M. (2021) Disentangling diverse responses to climate change among global marine ecosystem models. Progress in Oceanography, 198, 102659.

Lindmark, M., Audzijonyte, A., Blanchard, J. & Gårdmark, A. (2021) Bottom-up and top-down effects of temperature on body growth, population size spectra and yield in a size-structured food web. bioRxiv,

Lotze, H.K., Tittensor, D.P., Bryndum-Buchholz, A., Eddy, T.D., Cheung, W.W.L., Galbraith, E.D., Barange, M., Barrier, N., Bianchi, D., Blanchard, J.L., Bopp, L., Buchner, M., Bulman, C.M., Carozza, D.A., Christensen, V., Coll, M., Dunne, J.P., Fulton, E.A., Jennings, S., Jones, M.C., Mackinson, S., Maury, O., Niiranen, S., Oliveros-Ramos, R., Roy, T., Fernandes, J.A., Schewe, J., Shin, Y.J., Silva, T.A.M., Steenbeek, J., Stock, C.A., Verley, P., Volkholz, J., Walker, N.D. & Worm, B. (2019) Global ensemble projections reveal trophic amplification of ocean biomass declines with climate change. Proc Natl Acad Sci U S A, 116, 12907-12912.

Oliveros-Ramos, R., Verley, P., Echevin, V. & Shin, Y.-J. (2017) A sequential approach to calibrate ecosystem models with multiple time series data. Progress in Oceanography, 151, 227-244.

Spence, M.A., Blackwell, P.G., Blanchard, J.L. & Shuter, B. (2016) Parameter uncertainty of a dynamic multispecies size spectrum model. Canadian Journal of Fisheries and Aquatic Sciences, 73, 589-597.

Steenbeek, J., Buszowski, J., Chagaris, D., Christensen, V., Coll, M., Fulton, E.A., Katsanevakis, S., Lewis, K.A., Mazaris, A.D. & Macias, D. (2021) Making spatial-temporal marine ecosystem modelling better–a perspective. Environmental Modelling & Software, 145, 105209.

Tittensor, D.P., Novaglio, C., Harrison, C.S., Heneghan, R.F., Barrier, N., Bianchi, D., Bopp, L., Bryndum-Buchholz, A., Britten, G.L. & Büchner, M. (2021) Next-generation ensemble projections reveal higher climate risks for marine ecosystems. Nature climate change, 11, 973-981.

Wootton, H.F., Morrongiello, J.R., Schmitt, T. & Audzijonyte, A. (2022) Smaller adult fish size in warmer water is not explained by elevated metabolism. Ecology Letters, 25, 1177-1188.

Footnote: *Additional thoughts about physiological responses to temperature*

In our study we use a simplified and general approach of temperature effect on physiological rates, such as suggested by the Metabolic Theory of Ecology (Brown 2004). While MTE fails to make accurate predictions in specific situations, it provides a strong general framework, which, to our knowledge, has not yet been convincingly replaced. In most cases, physiological responses to temperature do involve some degree of rate acceleration (either directly as a physiological response or indirectly as a response to community reorganisation), at least in cases where temperature is unlikely to exceed species thermal optima, as could be expected in many cool water temperate systems. Intergenerational acclimation may cancel some or all of this metabolism increase, but the scale at which this acclimation occurs is unclear. For example, the study by the lead author in (Wootton *et al.*, 2022) showed that after four experimental generations baseline metabolic rates of zebrafish did not differ between 26C and 30C experimental temperatures. However, the authors pointed out that these findings only apply to a population with stable mortality conditions and should not be extrapolated to real communities, where temperature is also likely to drive changes in mortality and may cause indirect selection for increased metabolism, reported in in large scale spatial comparisons. Given the complexity and uncertainty of species and trait specific responses, assuming the same acceleration across species and traits currently is the most robust approach in a study that aims to compare physiological impacts versus impacts of benthic and pelagic primary production changes.
