# Supplementary material for: Changes in sea floor productivity are crucial to understanding the impact of climate change in temperate coastal ecosystems according to a new size-based model
Source: PLoS Biol. 2023 Dec 11;21(12):e3002392. doi: 10.1371/journal.pbio.3002392 (PMC10712853; doi:10.1371/journal.pbio.3002392)
Supplement: Attachment — Submitted filename: TasmModel_ResponseToReview2.docx [file pbio.3002392.s003.docx]

Dear Editors

We are submitting a revised version of the manuscript “A new size-based model highlights the importance of benthic production changes for understanding climate change impacts on a temperate coastal ecosystem” number PBIOLOGY-D-22-01984R2 for your consideration to be published in PLOS Biology. We thank the two reviewers for their comments and provide detailed response to reviewer 3 comments. We have also now created an official Zenodo repository release (with DOI) for all the code used in the model development and analyses (included in the publication). Note – line numbers refer to the document with track changes enabled.

We hope you now find our manuscript suitable for publication in PLOS Biology.

Yours sincerely on behalf of all co-authors

Asta Audzijonyte and Gustav Delius

*Reviewer #3:
[identifies himself as Diego Barneche]

I was invited to provide an additional independent assessment on the work by Audzijonyte et al. The authors used a physiologically structured food web model (a multi-species size spectrum model) to simulate how temperature and primary production from two different sources (benthic and planktonic) might affect a multitude of responses in the fish community, e.g., biomass, yield, average size of individuals. In particular, the authors were interested in evaluating the relative contribution of these variables to the responses based on a benchmark equilibrium state.
I have been given access to the previous round of reviews as well as the authors' rebuttal. I think the authors have done a good job at addressing the previous set of comments, and I agree with them that this is a timely contribution to the field. The model makes advances compared to previous ones, and it is (as much as realistically possible) well parametrised based on a unique long-term monitoring dataset from a region of relevance where warming effects have already been manifesting strongly. In my opinion, the key here is to make sure that the language reflects well the simulation nature of this study; and the authors certainly did a great job at clarifying that in this round. Moreover, the authors have provided an objective approach to evaluating parameter uncertainty, and have been explicit about the limitations of their approach (lines 459-465).
I do think though that some questions might be further explored in this paper. In my comments below, I will focus on some modelling questions (sensitivity analyses), overall suggestions to further clarify the text and to expand the discussion. I hope the authors find my comments useful.*
Thank you.

*\## Abstract
Here, the language could be improved to avoid confusion and be more explicit about the simulation nature of the study. Words like "evaluate", "identify", "drivers" give the impression that this is a data-driven predictive model. I make specific suggestions below. The conclusion sentence between lines 40-42 is not novel nor surprising. To me, to last two sentences of the abstract highlight well the novelty and relevance of the study.*

Thank you, agreed and corrected accordingly.  *\## Introduction
- Lines 127-129: This question 1) came (in part) as a surprise because the introduction does not articular how changes in food source will affect average size of individuals. Do you mean in similar ways as to a temperature-size rule effect? There's much better understanding of how temperature will affect size, but much less evidence with regards to the effect of overall food supply. This is generally thought to have a direct effect on overall population abundance, not average size of individuals. Whatever the mechanism, I think it would be important to articulate this expectation in the introduction.*

The individual level mechanism that these physiologically structured food web models are based on is from the long-established principles of bioenergetics, where all energy acquired through the ingestion of food is either used in metabolic processes, deposited as new body tissue (growth or energy gain) or lost as wastes in faeces or by excretion (Jobling 1994. Fish bioenergetics). Growth is of course also influenced by many other factors, only some of which we consider (temperature and competition of food). We have added a sentence and refences in the introduction, to better articulate our expectations (line 61 in the tracked changes document):

*“*Through the long-established principles of bioenergetics, we expect that changes in food availability (ultimately through changes in primary producers at the base of food webs) and temperature will both affect growth and size structure of fish populations (Jobling 1994). These changes in turn, through size-based food web interactions, are expected to alter mortality and reproduction, affecting population abundance and community structure (Andersen 2019).”

*\## Methods:
- Maybe it is an issue of pdf resolution, but the version of fig 1 available to me makes it really hard to visualise the red circles on the map. Is there a better way to clarify this? Perhaps by adding a third zoomed layer?*

A third zoomed layer is likely to make the figure too crowded and the exact position of the survey sites is not essential here as the model is focused on the entire south-east Tasmanian area. We have currently left the figure as it is, as it is likely to reproduce a lot better in print compared to the reviewer pdf version. But we are happy to modify it further, if requested.

*- Lines 167-172: I would argue that some top predators can be overestimated in reef visual surveys on a per-unit-area basis. As in, they likely obtain their energy sources from an area that is much larger (100s km) than the relatively smaller, typical survey areas (100-500 m2). How did the authors deal with that? An explanation would be well placed between lines 167-172.*

Thank you, this is now clarified in lines 172:

“To account for the fact that abundance of mobile larger predators (marine mammals, tunas, mobile sharks, pelagic predatory fishes, birds) may not be correctly reflected in underwater visual surveys on reef habitats, we did not use survey data for predator abundance, but included a general large predator species to represent the unaccounted predation. This general predator was assumed to grow to a maximum of 5 kg, which is an approximate average across birds, commonly observed mackerel and other medium sized pelagic fish species (maximum size of 2-3kg), but also occasional and much larger sharks and mammals. The predator abundance was chosen based on general expert knowledge and we also assessed sensitivity of model outcomes to this assumption by removing the predator completely and repeating the simulations (Supplementary material section 2.4).”

We found that the overall results in terms of biomass, yield and mean size changes across species and functional groups remained very similar.

*- Are lobsters and urchins being given unique importance because of commercial value, or because they are the main invertebrate prey in fish diets in Tasmania, or simply because they're the only invertebrate species that can be parametrised in terms of size? What would happen to the model outputs if urchins and lobsters were modelled as being part of the "background resource" like all other invertebrates? I think this is an important question that needs to be addressed in the appendix because it will speak to the robustness of simulation findings conditioned on model parametrisation an assumptions made for the base of the food chain.*

We now clarify this inclusion in line 183:

“Urchins and lobsters were included as separate functional groups because they are very abundant in the ecosystem (urchins are the most abundant group, see Table S1), they play important role as herbivores and predators, which would not be resolved if these groups were treated as a part of benthic resource spectrum, and because they reach sizes (Table 1) that are considerably larger than the maximum size of the benthic resource spectrum (5g, Table S2).”

We note, that benthic (or any other) resource is not interacting with other groups, but just provides food.

The question about the selection of functional groups in theory could be applied to some other groups as well, e.g. what would happen if the smallest planktivorous fish (hulafish) was treated as a part of the plankton spectrum? Selection of functional groups in multi-species models is often an arbitrary based on the main research question and species of interest, but we emphasise that in this study we consciously made an effort to remove this subjectivity by developing criteria of relative species abundance and frequency of occurrence to decide which species, from all observed species, should be to be included in the model. To our knowledge this is one of the first models that aims to provide this objectivity and this was possible because underwater surveys provide information about all observed fish species.

*- Is fishing mortality size dependent in the MSSS (I suspect not)? If not, a justification needs to be included because many fishery species have size-dependent fishing (as well as natural) mortality.*
Fishing mortality is size selective. A detailed explanation of model dynamics and equations is provided in the supplementary material, but we expanded the paragraph about model description, to reduce confusion (lines 231-250).

*- Lines 358-362: I would encourage the authors to consider an alternative approach to inclusion of temperature effects on the model. Daily CMIP6-derived SST time series (RCP8.5) for the region can be downloaded from NCI-ESGF, at least until 2050 (*[*https://pcmdi.llnl.gov/CMIP6/ArchiveStatistics/esgf_data_holdings/*](https://protect-au.mimecast.com/s/u079CYWL4BsLEVv2MsrmoPg?domain=pcmdi.llnl.gov)*). If the idea is to create as much realism as possible, why not use that rather than a fixed end-point temperature prediction?*

We have emphasised that our model does not aim to be realistic, as we clearly simplify many climate change impacts. We have explored relative impacts of a specific and fixed temperature change versus a specific and fixed primary production change in plankton and benthic resources. These specific and fixed values of climate change scenario allow us to decompose the role of temperature, food and species interactions. We show that even with simple 2C temperature change emergent responses are different across species and vary depending on the trophic group. Further, earth system models (ESMs) also make a lot of assumptions - free running ESMs used in climate projections are not constrained by observations and using these projections alone would not make sense. Moreover, for each temperature scenario we would then need realistic values of the primary production and benthic production to go along. CMIP6 models do not include benthic primary production, and even the primary production is uncertain and not available for the spatial scale relevant for coastal reef communities. Most importantly, we do not explore temporal dynamics of the system, but equilibrium conditions of baseline (not heated) and 2C warmer ecosystem. This is a common approach in simulation studies, which focus on alternative stable states of a system under two alternative scenarios. Including a specific temperature scenario time series therefore is not meaningful here. We have added a sentence to clarify this point (Line 384).

“Moreover, we do not explore temporal evolution of the model, but just equilibrium conditions under alternative physiological and food availability states.”

*## Results & Discussion
- I cannot help but wonder what the high-level expectation of the proposed scenarios will be, i.e., completely ignoring all of the complex mechanisms that compose the MSSS. For example, if overall per-capita metabolic demand is to increase by ~20% (a consequence of the 0.63 eV and a +2.5˚C change in temperature), and resource abundance changes by X fold (changes with scenarios), then what is the naive expectation of overall change in community biomass (i.e. assume no change in size structure)? I guess this would be extremely useful to help the reader appreciate how much the added mechanisms in the MSSS influence the departure from such a naive expectation. If the overall ratio between autotrophic and heterotrophic biomass doesn't change in the model, then I would expect the model to yield results comparable to the naive expectation. This would make a really cool expansion of Figure 4. There could a 5th panel containing the community-level changes, and another dashed line showing the naive expectation.*

This is an interesting question, but we are not sure how this could even be answered, or what exactly the reviewer is asking us to do. From what we understand the reviewer is asking us to compare our results to a model without multi-specie size-based interactions, such as a biomass pool model in Ecosim? It was not really clear to use whether reviewer expected different fish groups to be represented as different biomass pools and without MSSS mechanisms, or whether all consumers should have one biomass pool. Either way, we are not sure such a comparison makes a lot of sense because the two models make such different assumptions about the underlying ecosystem processes. A biomass pool model would have to make a lot of additional assumptions on how different sizes should be represented in the biomass pool and how they should interact with other sizes somehow represented in other biomass pools. Moreover, the question is not about simple increase in metabolic demand versus changes in resource abundance. In our simulations temperature affected all vital rates, including size dependent food intake and mortality. We believe we have partly addressed this question by comparing outcomes of simulations with and without physiological changes. Otherwise, we are not sure how this question should be tackled.

*- Lines 740-749: It would be good to state whether maximum size is present in the mizer framework. If so, is it allowed to evolve? I'm not sure if this component is captured in the explanation given between lines 765-784.*

Maximum size is present in mizer framework and is the key species parameter. In fact this is the only species parameter that we present in the main Table 1 (all other parameters are given in the supplementary materials). The maximum size determines energy allocation between growth and reproduction and it does not evolve, however realised growth curves and size distributions can change through time and are emergent. Adding evolution into this model would be an entirely new study with an entirely new set of questions and results – see, for example, Forestier et al. 2020 (<https://onlinelibrary.wiley.com/doi/full/10.1002/ece3.6995>), where maturation size was allowed to evolve and where we explored outcomes of this evolution on species interactions.

*Otherwise how are changes in mean size emerging? The authors' explanation, although consistent with empirical data (lines 750-764), seems to imply that baseline conditions wouldn't allow individuals to achieve their maximum potential (size wise). My understanding is that individuals generally do not achieve their maximum sizes because they die early (so it's more about time rather than resource abundance). Generally more food would allow more individuals over time, but not necessarily bigger individuals. Some discussion around the relevance of this to wider ecological literature would be helpful.*

Changes in energy intake (through food availability and feeding rates), energy expenditure (through metabolism) and mortality (through emergent size based predation) will affect numbers of individuals in each size class. Size structure is an emergent property of the model. Therefore, the mean size is also an emergent property as it calculates the average individual size across the size classes. More food means that individuals growth faster, but it could also mean that predation rates increase. This could lead to either increasing or decreasing mean sizes, depending on the interplay of growth and mortality on different size classes. This is now also explained in the main text (line 241), in the paragraph explaining the model structure and emergent properties.  *\## Additional thoughts:
- What happens if the model does not have the additional 5 kg predator?*
See the answer above, we ran additional simulations (now included in the supplementary results) and found that the main conclusions remain unchanged. This is now also added to the Results (line 607).

“When the general predator species was removed from the model (to explore model sensitivity to the assumptions about its abundance) the overall results about the changes in biomasses, yields and mean sizes across the simulated scenarios remained very similar (Supplementary material section 2.4).”

*- How does the model deal with detrital recycling? It can be a major source of energy in the system (e.g. Brandl et al. 2019 10.1126/science.aav3384).*
We do not have detrital recycling in this model. This aspect has been explored in other simpler models, for example Blanchard et al. 2009, 2011(<https://link.springer.com/article/10.1007/s12080-010-0078-9> and <https://besjournals.onlinelibrary.wiley.com/doi/10.1111/j.1365-2656.2008.01466.x>). And for reefs see Rogers et al. 2018. We agree the paper by Brandl is interesting and it could be an area for future work. We opted to use the empirical size-distribution of benthic invertebrates, as the data required to parameterised detrital dynamics in the above models was not available at the scale of this study.

*- Are current conditions under equilibrium? There's 30 years worth of calibration data—what does this reveal in terms of equilibrium?*
Arguably, natural ecosystems are never in equilibrium and we acknowledge that by allowing model biomasses to be within 4x range from the average biomasses for the calibration period (1990s) (line 482, already explained in the original submission).

“Each run was rejected if, at the end of the 150-year simulation, biomasses of at least one species were 4 times smaller or larger than the average biomasses observed in underwater surveys. We allowed average biomasses to range 4 times because across year variation in underwater surveys also showed similar levels of variability (Fig. S1)”

Allowing 4x time variation in biomasses allows the system to be in a range of alternative states and does not assume equilibrium. Most models aim to reproduce observed biomasses within a small range of the observed average or as closely as possible. In our model we use 29 alternative parameter combinations to reflect alternative plausible ecosystem states. We have added a further explanation to highlight that this wide biomass variation allowed in our parametrisation aims to reflect inherent non-equilibrium conditions of the ecosystem (line 485).

“Allowing biomass to vary extensively also better reflects the fact that natural ecosystems are never truly in an equilibrium state and species biomasses naturally fluctuate. Our alternative parameter combinations therefore reflect a range of possible ecosystem states”.

*- The authors did a really good job at stating the expectations/generalisations from their study while acknowledging major limitations. There are a few missing processes, though, which I think would be important to mention explicitly. For example, how are nutrient regimes expected to change over time in SE Tasmania (see, e.g., Moore et al. 2019 10.1126/science.aao6379)? This could have major implications to primary production.*

We are not sure we understand this comment. The entire point of the study is to explore how changes in primary pelagic and benthic production (likely driven by nutrient changes, but also other factors) might affect the ecosystem. So the answer is – yes, the nutrient regimes are likely to change, but predictions about expected changes in primary production are highly uncertain and suggest both increases and decreases. We was described in Methods (line 332-339):

“For example, it is generally expected that increasing temperature favours smaller body size and that higher temperatures will lead to steeper background resource size spectra (Reuman et al. 2008, Polovina and Woodworth 2012, Peter and Sommer 2013, Tittensor et al. 2021) (see Supplement). Yet empirical evidence shows that changes in temperature also affect nutrient availability and grazing pressure (Mazurkiewicz et al. 2020, Pomati et al. 2020), that benthic resource size spectra may be insensitive to temperature (Mazurkiewicz et al. 2020, Heather et al. 2021), and field data from the last 10 years shows increasing zooplankton abundance off Maria Island (Everett et al. 2020). We therefore explored a range of resource change scenarios, with plankton or benthos abundance ($\kappa$) and slopes (λ) increasing or decreasing independently (Table 2).”

It is also discussed further in Supplementary material sections 1.7-1.8.

*The last paragraph briefly mentions species reorganisation, but it does not discuss the potential effects of larval connectivity coming from the north. Finally, another important aspect would be species metabolic adaptation which can happen quite fast, particularly in phytoplankton, and the prognosis does not look encouraging (see e.g. Barton et al. 2020 10.1111/ele.13469). From their scenarios, which one(s) would be most consistent with these predictions of nutrient and yield in phytoplankton primary production? This would be an important aspect to highlight.*

Adaptation and acclimation are not included in the model and this is highlighted as a limitation. This applies both for phytoplankton and fish. We thank the reviewer for suggesting this reference and we also include it in our study; although we do not specifically model metabolic rates of resource spectra (e.g. plankton metabolism). The question of acclimation remains a big challenge of climate change predictions in ecosystem models and an important area of future exploration. We have added a few more sentences to the discussion to highlight this challenge (Line 778).  *\# Specific comments:
words in [] mean an addition, words in ** mean a deletion

\## Overall
- Standardise the use of multi-species. In some parts of the text it has been written as "multispecies" (no hyphen).*

Fixed, thank you. We now use multispecies throughout.  *\## Abstract
- Line 30: "... specifically designed to *evaluate* [simulate] potential ..."
- Line 31: "... and *identify* [calculate] ..."
- Line 32: "... these *drivers* [variables] ..."*
All done

*## Introduction
- Line 68: Consider citing De Roos & Persson (2001) here.*

Done *- Lines 89-92: This is an important statement that needs to be backed up by a reference.*

Reference added – Liu et al. 2021

*- Line 103: "... *substrate* [substratum] ..."*

Done  *- Line 112: "... *refugia* [refuge] ..."*

This sentence refers to multiple refugia, not one refuge. Left unchanged  *\## Methods
- Line 215: But was growth efficiency used in your model or not? This is likely a temperature dependent process, and theoretically said dependence might affect the slope of the size spectrum.*

This is now clarified (line 236) – explaining that growth efficiency is applied here but not usually used in other mizer applications. The growth efficiency in our formulation applies specifically to the conversion of assimilated energy to growth, once metabolic costs have already been accounted for. This is different from some other notions of growth efficiency, which refer to the conversion of energy from intake to growth, and which includes assimilation efficiency and metabolism. In this broader range growth efficiency is likely to be sensitive to temperature (through changes in metabolism) and this is accounted for in our model, because metabolism (modelled explicitly) is dependent on temperature. We have not modified text further here, as these different interpretations of growth efficiency are not relevant here and would introduce confusion.

*- Line 291: Is this macroalgal biomass density typical in SE Tasmania? This needs explicit mention in the text.*

Macroalgal biomass in 1990s was very high and unlikely to be limited. It is not clear what proportion of the biomass in real ecosystems is actually available for feeding (in terms of nutritional value). Model based biomasses are all available for herbivores and have the same energetic value, so direct comparisons are difficult. The main point for this selection of parameters is to ensure that high feeding levels for herbivores (i.e. no food limitation). This is clarified in line 313, and we note that original submission explained that modelled macroalgal biomasses do not fully represent the large kelp stands (line 312).

*- Line 302: Please cite Yvon-Durocher et al. 2011 (10.1111/j.1365-2486.2010.02321.x)-this is data based evidence that warming steepens the size spectrum slope.*

Done.  *- Line 342: That equation is not actually used in Brown et al. 2004. It is proposed (equation 3) in Gillooly et al. 2001 (10.1126/science.1061967). Please swap the citation to the correct one.*

Done.  *- Line 347: Please provide a reference for the 0.63 eV choice. Marine phytoplankton can have much steeper temperature dependence than heterotrophic organisms, particularly those from cooler water regimes. Please see Barton & Yvon-Durocher 2019 (10.1002/lno.11170).*

Please note that the scaling of vital rates does not apply to phytoplankton or benthos here, only to the physiological processes explicitly modelled in functional groups (fish, urchins and lobsters). Changes in plankton, benthos and macroalgal resource physiology are reflected through overall changes in their abundance (kappa) or size spectrum slope (lambda). We provide a reference to Lindmark et al. 2022, which gives a review of activation energies in different fish species for intake and metabolism and finds that the averages (0.62 and 0.69, but wide uncertainty ranges) are close to commonly used value of 0.63.  *\## Results & Discussion
- Fig 3: What are the units across the x axes?*

This is now corrected and explains that the units are in % difference.  *- Lines 624-627: I would suggest removing this sentence entirely, it does not fit well with the ecological relevance of the finding, which is the main topic of the paragraph.*

Done.  *- Lines 627-629: I would cite Naumann et al. 2013 (10.1371/journal.pone.0082923), who suggest that benthic primary production can in fact be substantial. This helps the cause.*
Thank you, done.
